# Ferroelectric Properties of Bilayer MoS_2_/WS_2_ Heterostructure Modulated by Twist Angle

**DOI:** 10.1002/advs.202513738

**Published:** 2025-10-08

**Authors:** Liyao Wang, Zhuopeng Xia, Xiaoyao Sun, Shiyao Xu, Guodong Sun, Yingying Zheng, Zhen Zhan, Enlong Li, Songhua Cai, Yuan Zhang, Jinzhu Zhao, Wenwu Li, Shuoguo Yuan

**Affiliations:** ^1^ Faculty of Materials Science and Chemistry China University of Geosciences Wuhan 430074 P. R. China; ^2^ Guangdong Provincial Key Laboratory of Quantum Engineering and Quantum Materials School of Physics and Guangdong‐Hong Kong Joint Laboratory of Quantum Matter South China Normal University Guangzhou 510006 P. R. China; ^3^ Department of Materials Science and Engineering Southern University of Science and Technology Shenzhen 518055 P. R. China; ^4^ Department of Applied Physics The Hong Kong Polytechnic University Hong Kong 999077 P. R. China; ^5^ Shanghai Frontiers Science Research Base of Intelligent Optoelectronics and Perception Institute of Optoelectronics College of Future Information Technology Fudan University Shanghai 200433 P. R. China; ^6^ Center for Computational Science and Engineering Southern University of Science and Technology Shenzhen 518055 P. R. China; ^7^ National Laboratory of Solid State Microstructures Nanjing University Nanjing 210093 P. R. China; ^8^ Shenzhen Institute China University of Geosciences Shenzhen 518057 P. R. China

**Keywords:** 2D materials, ferroelectricity, field‐effect transistors, MoS_2_/WS_2_ heterostructure, twist angles

## Abstract

The emergence of sliding ferroelectricity is found in non‐ferroelectric two‐dimensional materials, which brings novel ferroelectric phenomena and expands the potential for advancing ferroelectric devices. Experimental studies have largely focused on sliding ferroelectricity with fixed twist angles owing to the limitations of preparation methods with controlled angles. However, how to modulate the ferroelectric properties in the sliding materials is still challenging. In this work, the out‐of‐plane ferroelectric properties of typical bilayer MoS_2_/WS_2_ heterostructure are reported by precisely controlling twist angles. The experimental results demonstrate that the second‐harmonic generation response, indicative of symmetry breaking, decreases as the twist angle increases. In addition, the switching voltage of ferroelectric polarization exhibits the opposite trend with increasing the twist angle. According to experimental studies and theoretical calculations, the tunability of ferroelectric properties arises from the distortion of polar symmetry regions induced by Moiré patterns at different twist angles. Furthermore, the ferroelectric semiconductor field‐effect transistors yield the twist angles dependent electrical properties, achieving a large ferroelectric memory window of ≈14 V. The study opens the door to significantly modulating the sliding ferroelectricity via designing twist angles, which will enrich the framework of twistronics and expand the promising applications in the emerging sliding ferroelectric devices.

## Graphical abstract

The study reports the precise control of interlayer twist angles in the bilayer MoS_2_/WS_2_ heterostructure. The ferroelectric properties of MoS_2_/WS_2_ heterostructure can be significantly modulated by designing twist angles, resulting from the distortion of polar symmetric regions induced by Moiré patterns. The study opens the door to manipulate sliding ferroelectric properties, which will bring emerging ferroelectric twistronics devices.

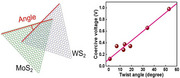

## 1 Introduction

Two‐dimensional (2D) ferroelectric materials have attracted tremendous attention thanks to the atomic scale, no dangling bonds, and incorporation with semiconductor materials, which have promising applications for ferroelectric memory, transistor, and neuromorphic computing.^[^ ^1^, ^2^ ^]^ A large number of 2D ferroelectric materials have been theoretically predicted, but a few 2D materials including CuInP_2_S_6_, MoTe_2_, and In_2_Se_3_ have been experimentally reported to exhibit intrinsic ferroelectricity.^[^ ^3^, ^4^, ^5^ ^]^ Nevertheless, the majority of 2D materials are unable to spontaneously form ferroelectric polarizations due to the absence of the requisite specific crystal structures and electronic configurations necessary for ferroelectricity. Twist engineering enables the emergence of sliding ferroelectricity in inherently non‐ferroelectric 2D materials, unveiling unprecedented physical behaviors.^[^ ^6^ ^]^ The ferroelectricity observed in this twisted bilayer can be attributed primarily to the formation of Moiré lattice and interlayer sliding. Additionally, it is closely associated with interface engineering and energy band reconstruction.^[^ ^7^, ^8^ ^]^ The breaking of symmetry by twisting has prompted a significant expansion in the study of ferroelectricity of numerous 2D materials.

Recent studies have demonstrated that twist engineering can produce unique physical properties of 2D materials. This encompasses the interlayer binding energy and chiral vortex domains of MoS_2_ homojunctions, reflected in the PL spectra, exciton energies, and Raman peak positions, all of which demonstrate a pronounced dependence on the twisting angle.^[^ ^9^ ^]^ The exciton transition strengths and energies in WSe_2_/MoSe_2_ heterostructure, tunability of the spin texture in WSe_2_/graphene heterostructures,^[^ ^10^, ^11^ ^]^ and the dipolar excitons in black phosphorus have been demonstrated to undergo alteration with the interlayer twisting angle.^[^ ^12^ ^]^ Furthermore, interlayer twisting has been employed in the exploration of ferroelectricity. Examples of this include the construction of a bulk nonpolar 2D material, boron nitride and graphene, by twisting to make it ferroelectric, etc.^[^ ^13^ ^]^ Previous studies have mainly concentrated on sliding ferroelectricity with fixed angles due to the limitations of preparation methods with controlled angles. However, it is difficult to achieve the modulation of ferroelectric properties in sliding materials.

In this work, we report that the ferroelectric properties of bilayer MoS_2_/WS_2_ heterostructure can be modulated via varying twisting angles over a symmetry period. The twisted MoS_2_/WS_2_ heterostructure has been constructed by chemical vapor deposition (CVD) growth and transfer method. The symmetry and ferroelectric behavior of MoS_2_/WS_2_ heterostructure have been measured by second‐harmonic generation (SHG) and piezoresponse force microscopy (PFM). Experiment results indicate that the ferroelectric properties can be modulated by twisting angles. The theoretical calculations serve to elucidate the physics mechanism of twisting angle‐dependent ferroelectric properties. Furthermore, the ferroelectric semiconductor field‐effect transistors (FeS‐FETs) have been constructed with the aforementioned twisted van der Waals heterostructure, which exhibit twist angles dependence of electrical properties, achieving large memory window (MW) and high mobility, which is higher than previously reported untwisted heterostructures.^[^ ^14^, ^15^ ^]^

## 2 Results and Discussion

### 2.1 CVD Growth and Structural Characterization of Twisted MoS_2_/WS_2_ Heterostructure

**Figure 1:**
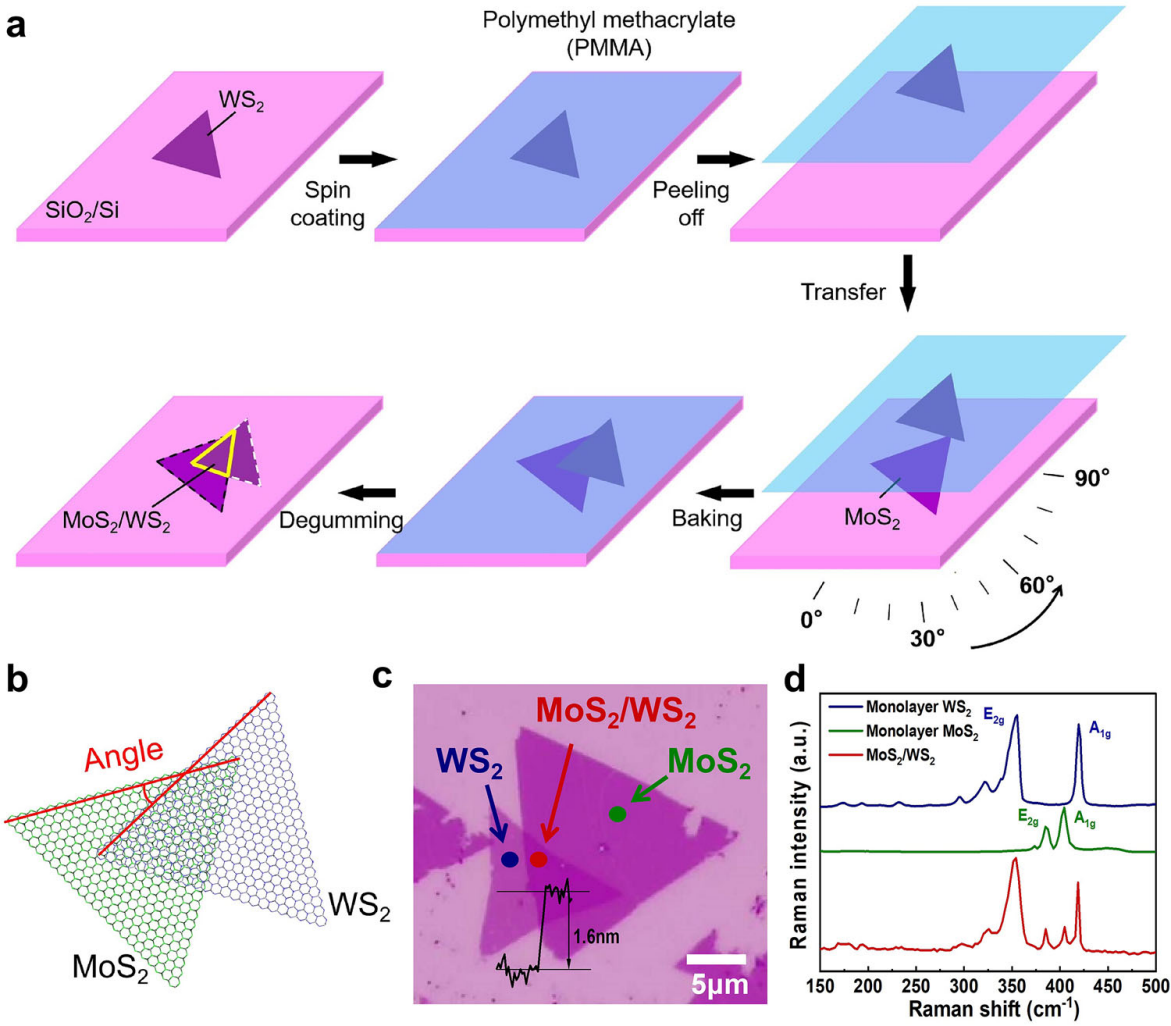
Characterization of twisted MoS_2_/WS_2_ heterostructure. a) Schematic diagram of the preparation of twisted MoS_2_/WS_2_. b) Schematic diagram of atomic structure for twisted MoS_2_/WS_2_. The red line represents the judgment method for angle calibration. c) Optical microscopy image of MoS_2_ and WS_2_ monolayers, as well as MoS_2_/WS_2_, and the inset shows the height curve of bilayer MoS_2_/WS_2_. d) Raman spectra of monolayer MoS_2_, monolayer WS_2_, and MoS_2_/WS_2_.

Due to the limitations of precise control twist angles of bilayer samples by the mechanical exfoliation method, the MoS_2_ and WS_2_ samples were grown by CVD, respectively. The high‐quality bilayer MoS_2_/WS_2_ heterostructures were employed to actively manipulate the interlayer twist angle via the transfer method, as illustrated in **Figure** **1**a. The specific growth parameters and post‐growth optical microscope images are presented in Figure S1 (Supporting Information). A schematic representation of the atomic structure of the MoS_2_/WS_2_ heterostructure is shown in Figure 1b. The MoS_2_ is located on the right side as the lower layer, the WS_2_ is depicted on the left side as the upper layer, and the central stacked section belongs to the MoS_2_/WS_2_ heterostructure. The red line illustrates the methodology employed to ascertain the stacking twist angle. The optical microscopy image of twisted bilayer MoS_2_/WS_2_ heterostructure is presented in Figure 1c. The atomic force microscopy (AFM) result shows that the thickness of bilayer MoS_2_/WS_2_ heterostructure is determined to be ≈1.6 nm, as illustrated in the inset. The Raman spectra of MoS_2_/WS_2_ heterostructure are presented in Figure 1d, which shows the typical in‐plane vibrational modes (E_2g_) and out‐of‐plane vibrational modes (A_1g_) of MoS_2_ and WS_2_ monolayers. The Raman modes of WS_2_ are located at 355 and 419 cm^−1^, while the Raman modes of MoS_2_ are located at 385 and 404 cm^−1^. The overlapping region corresponds to the MoS_2_/WS_2_ heterostructure. Furthermore, the interface of the MoS_2_/WS_2_ heterostructure has been characterized by scanning transmission electron microscopy and energy‐dispersive X‐ray spectroscopy, as presented in Figure S2 (Supporting Information). The clear interface has demonstrated the high quality of the heterostructure without the obvious stacking disorder.

### 2.2 SHG Response of Twisted MoS_2_/WS_2_ Heterostructure

**Figure 2:**
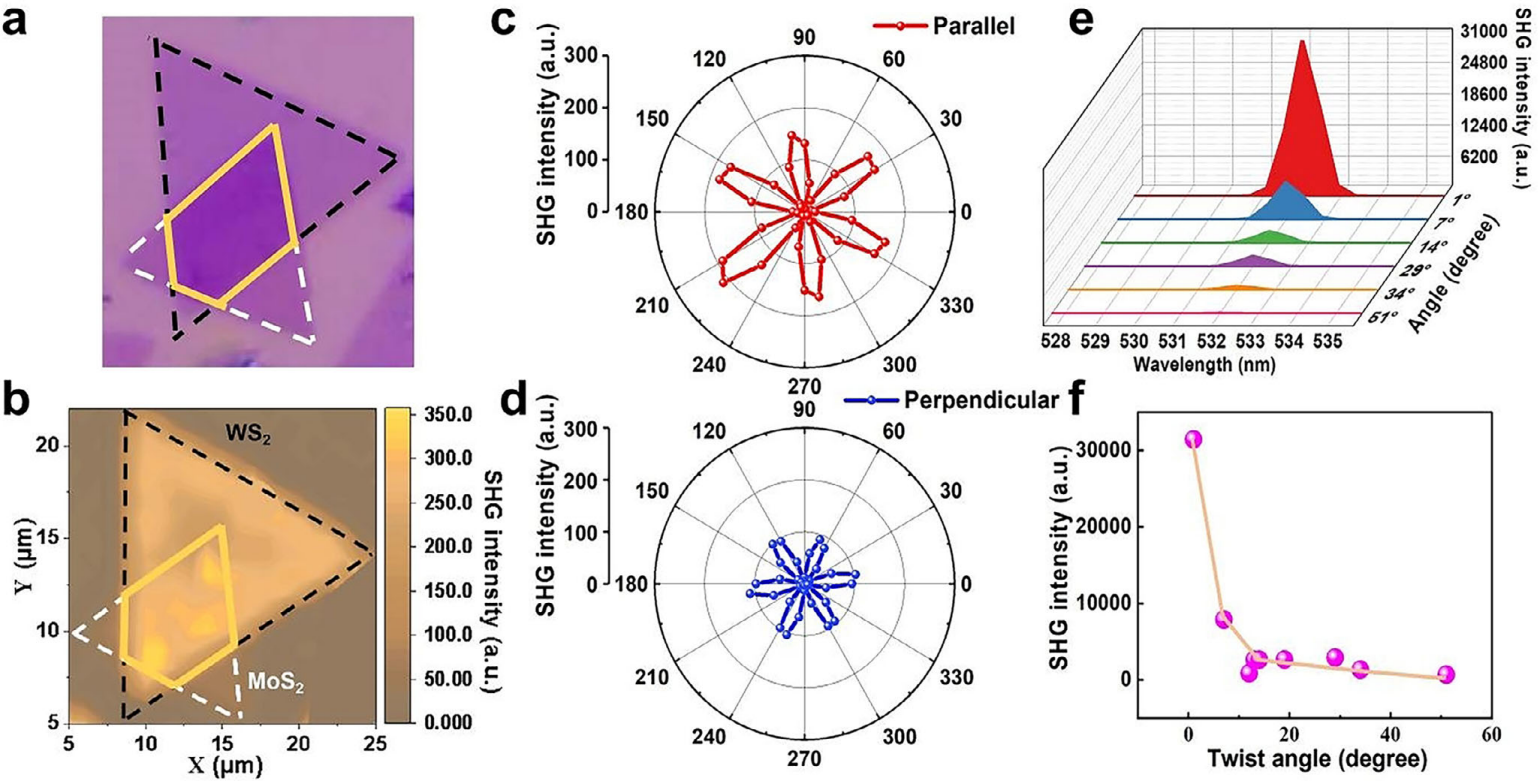
SHG characterizations of twisted MoS_2_/WS_2_ heterostructure. a) Optical microscope image of MoS_2_/WS_2_. b) Corresponding SHG mapping image of MoS_2_/WS_2_. c,d) Polarization SHG intensity of MoS_2_/WS_2_ under parallel (c) and perpendicular (d) polarization configurations, respectively. e) Using an excitation wavelength of 1064 nm, SHG intensity as a function of different twisting angles and wavelengths. f) SHG intensity with twisting angle variation.

Typically, SHG is capable of providing an accurate assessment of symmetry. **Figure** **2**a illustrates an optical microscope image of the MoS_2_/WS_2_ heterostructure at a twisting angle of 53°. The black shape represents the upper WS_2_ layer, white shape represents the lower MoS_2_ layer, and yellow box represents the stacking angle. Figure 2b shows the corresponding SHG mapping image. As evidenced by the color comparison, the stacked region of MoS_2_/WS_2_ heterostructure presents a more intense yellowish tone, which indicates that the strength of SHG response of the heterostructure is greater than that of the two individual materials. The polarization SHG signal under a representative parallel emission field, as depicted in Figure 2c, can be approximated by the equation *I* = *I*_0_ cos^2^3*θ*. Figure 2d shows the polarization SHG signal under the perpendicular emission field, which can be approximated by the equation *I* = *I*_0_ sin^2^3*θ*. In this context, *I*_0_ represents the maximum value of the SHG intensity, while *θ* denotes the azimuthal angle between the direction of the excitation field and crystal mirror. The two diagrams collectively demonstrate that the sixfold rotational symmetry of the SHG remains integral within the twisting region. The angular difference of 30° in parallel and perpendicular configurations of the heterostructure exhibits typical rhombic symmetry, confirming its non‐centrosymmetric structural characteristics, which fulfill the fundamental criterion for a material with ferroelectric properties.^[^ ^16^ ^]^ More results with the horizontal and vertical polarization SHG signals are shown in Figure S3 (Supporting Information). Figure 2e illustrates the intensity of SHG signal as a function of twisting angles, and Figure 2f shows the corresponding curve of the decreasing trend in SHG signal intensity with increasing twisting angles. The results taken at additional twisting angles are presented in Figure S4 (Supporting Information). According to SHG measurement, it is clearly shown that a strong twist angle dependence of SHG intensity, the maximal SHG intensity is detected at small interlayer twist angles. As the interlayer twist angle gradually increases, the SHG intensity decreases, and the corresponding degree of symmetry breaking gradually diminishes. This indirectly reflects the modulatory effect of the interlayer twist angle on structural symmetry of MoS_2_/WS_2_ heterostructure.

### 2.3 PFM Characterization of Twisted MoS_2_/WS_2_ Heterostructure

**Figure 3:**
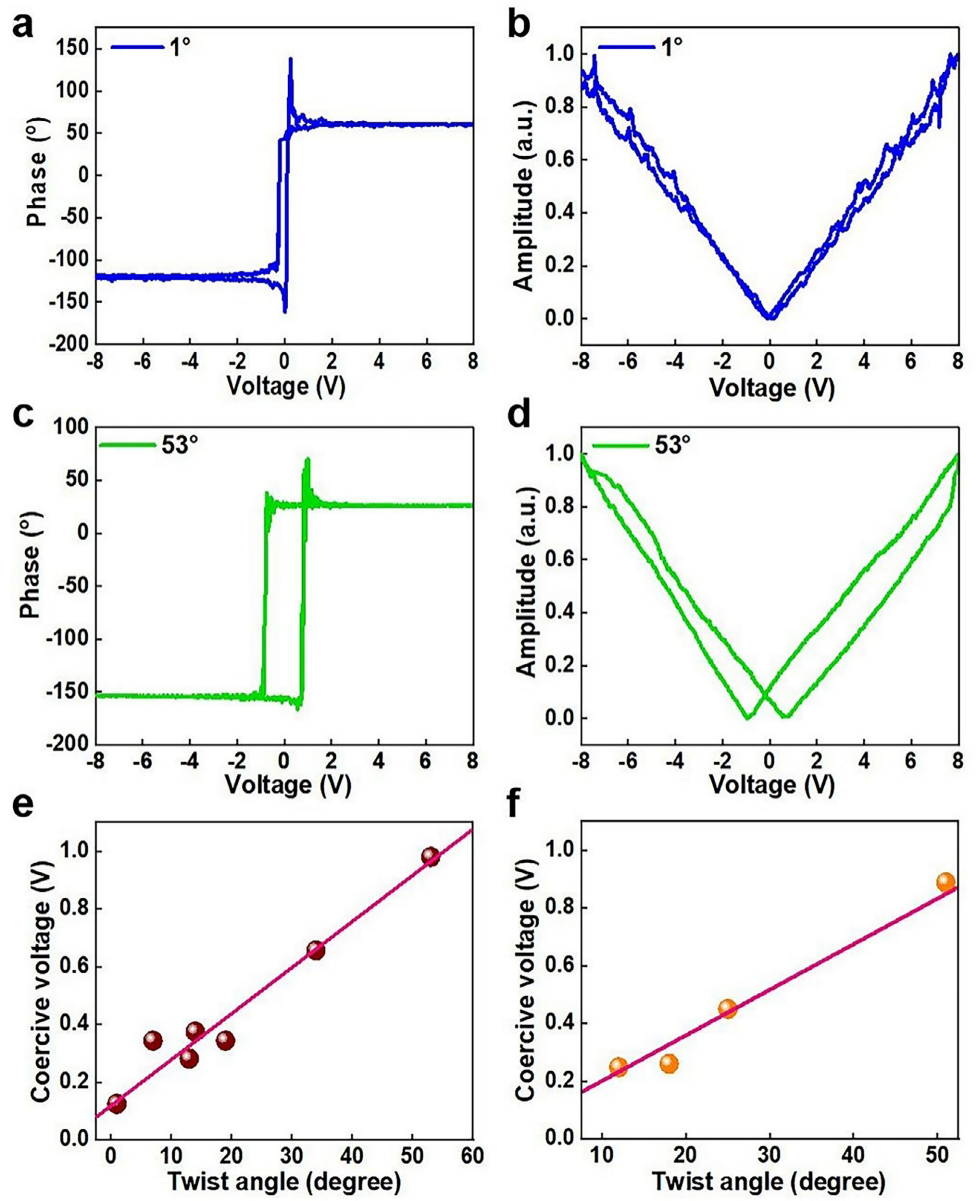
PFM measurement of twisted MoS_2_/WS_2_ heterostructure. a,b) PFM phase loop and amplitude loop of MoS_2_/WS_2_ with twist angle of 1°. c,d) PFM phase loop and amplitude loop of MoS_2_/WS_2_ with twist angle of 53°. e) Twist angle dependent coercive voltage values of MoS_2_/WS_2_. f) Reproducibility assessment of coercive voltage values of MoS_2_/WS_2_.

It has been shown that for single monolayer MoS_2_ and WS_2_, the inherent inversion symmetry prohibits the formation of ferroelectricity. However, when the two layers are stacked together and structurally twisted, the central inversion symmetry is disrupted, resulting in the emergence of switchable vertical polarization with sliding ferroelectricity.^[^ ^16^ ^]^ **Figure** **3**a–d present the vertical PFM phase and amplitude loops of MoS_2_/WS_2_ heterostructure at a twisting angle of 1° and 53°, which demonstrates the 180° phase change and typical butterfly‐shaped amplitude behavior. More PFM results with different twisting angles are presented in Figure S5 (Supporting Information). To rule out the charge effects, PFM characterizations have been performed after 72 hours, as shown in Figure S6 (Supporting Information), confirming the stability of the ferroelectric polarization state. These results demonstrate the out‐of‐plane ferroelectricity in the MoS_2_/WS_2_ heterostructure, which enables vertical polarization switching under applied voltage. The vertical PFM phase and amplitude hysteresis loops are asymmetrical with respect to zero bias as a consequence of the interfacial charges and internal electric fields.^[^ ^7^ ^]^ Subsequently, the ferroelectric polarization switching voltages (negative‐bias) for varying stacking angles are quantified, as illustrated in Figure 3e. The data shows an upward trend in coercive voltage as the twisting angles increase, which demonstrates that the ferroelectricity of MoS_2_/WS_2_ heterostructure can be modulated by twist angles. In addition, the ferroelectric polarization switching voltages using positive‐bias exhibits the same trend. To verify the accuracy of this trend, more twist angles have been selected for the repeatability measurement, as presented in Figure 3f. The coercive voltage gradually increases with the increase of interlayer twist angle, clearly determines that interlayer twist angle can affect the ferroelectric properties of MoS_2_/WS_2_ heterostructure.

### 2.4 Theoretical Calculation of Twisted MoS_2_/WS_2_ Heterostructure

**Figure 4:**
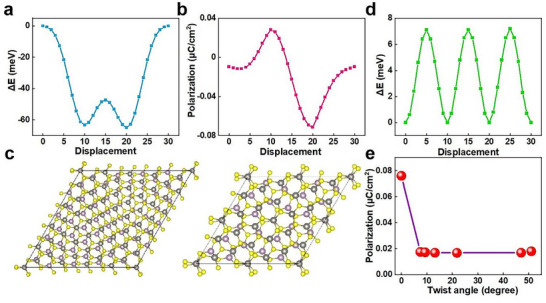
Theoretical calculation of ferroelectric properties of twisted MoS_2_/WS_2_ heterostructure. a,b) The case of AA‐stacking, the potential energy surface (a) and the evolution curve of the vertical polarization value (b) with sliding of the MoS_2_/WS_2_ heterostructure. c) Top view of Moiré superlattice with twist angle of 7° and 21° (purple, black, and yellow colors denote Mo atom, W atom, and S atom). d) Potential energy surface of the material during diagonal sliding with twist angle of 21°. e) Vertical ferroelectric polarization values for different twist angles.

To elucidate the physics mechanism of twisting angle‐dependent ferroelectric properties of MoS_2_/WS_2_ heterostructure, we perform first‐principles calculations to address the microscopic origin of ferroelectric properties. The lattice constants of monolayer MoS_2_ and WS_2_ are a = b = 3.167 Å and a = b = 3.174 Å, respectively. Both of them belong to the P‐6m2 layer group. After full structural relaxation, the lattice constant of the MoS_2_/WS_2_ bilayer heterostructure includes a = b = 3.168 Å. We first study the case for the untwisted AA stacking configuration (Figure S7, Supporting Information). To identify the ferroelectric migration path of the heterostructure under AA stacking, the polarization variation of the bilayer heterostructure has been investigated when it slides along the diagonal direction of the unit cell. As the potential energy curves reported, as shown in **Figure** **4**a,b, where dis0, dis10, dis20, and dis30 refer to sliding along the diagonal for 0, 1/3, 2/3, and 1 unit‐cell displacement, respectively, the minimum energy appears at the positions of 1/3 and 2/3 in the unit cell along the diagonal direction. The point with the most stable energy is the position where the bilayer slides by 2/3. In addition, the vertical polarization reverses its direction.

Since the constraint of the lattice symmetry, four different angles of the bilayer heterostructure are calculated, including 21°, 13°, 9°, and 7°, respectively. These four distinct rotational configurations require expansion to superlattices that are 7‐fold, 19‐fold, 37‐fold, and 61‐fold, respectively, to ensure the symmetry of the lattice is satisfied. Additionally, with smaller rotation angles, Moiré patterns become more pronounced (e.g., at 7°). All four structures exhibit the C3 point group symmetry. Figure 4c illustrates the atomic structure at a twist of 7° and 21° (different twisting angles of the atomic structure are shown in Figure S8, Supporting Information), and Figure 4d illustrates the potential energy surface for sliding at 21°. In the absence of a twisting angle, the vertical polarization attains its maximum value. With the increment of the twist angle, the magnitude of the vertical polarization is reduced. The data in Figure 4e shows that as the twisting angle increases, the trend of magnitude of vertical polarization gradually decreases; this feature is consistent with the SHG result. Furthermore, the kinetic barriers for polarization switching and the role of interlayer charge transfer are shown in Figure S9 (Supporting Information). With increasing twist angle, the charge asymmetry has gradually diminished, and the polarization component has concurrently decayed, which has been qualitatively consistent with the suppression of polarization induced by twisting.

Combining the information obtained from the above calculations, the twisting angle‐dependent ferroelectric polarization can be understood as follows. When the twisting is suppressed, the vertical polarization in bilayer MoS_2_/WS_2_ heterostructures is influenced by symmetry breaking. When the twisting angle becomes non‐zero, a super cell with Moiré pattern of the lattice formed. It should be noticed that the atomic structures and the local symmetry for part of the polar region will be distorted away from the ferroelectric untwisted configuration. The average polarization of such distorted local regions will be zero in lattice, while the percentage volume of such region will quickly increase along with the twist angle. This results in a gradual decrease in the polarization value over the range of twisting angles, with a tendency for the decrease and keeping an almost constant value after 7°. This constant value is similar to the reported ferroelectric polarization value, which is still suitable for device applications.^[^ ^17^, ^18^ ^]^ This correlation has been corroborated following comprehensive research, thereby providing a crucial foundation for the optimal design and performance regulation of materials.^[^ ^19^ ^]^

The ferroelectric switching voltage represents the minimum voltage to reverse the polarization direction of a ferroelectric material when subjected to an external electric field. The main mechanism is attributed to the nucleation and growth of new domains when polarization reversal is achieved.^[^ ^20^ ^]^ The magnitude of the switching voltage has been susceptible to numerous influencing factors, among which structural symmetry is a significant element. In materials with high symmetry, the weaker polarization intensity results in a relatively uniform charge distribution within the domains, which leads to a relatively difficult nucleation and growth process of new domains. Consequently, a higher applied electric field is required to provide sufficient energy to switch the direction of polarization. In contrast, in materials with low symmetry where the polarization intensity is stronger, a lower switching voltage is sufficient to reverse the polarization direction, which is consistent with our experimental results.^[^ ^21^, ^22^ ^]^

### 2.5 MoS_2_/WS_2_ Heterostructure Based FeS‐FETs

**Figure 5:**
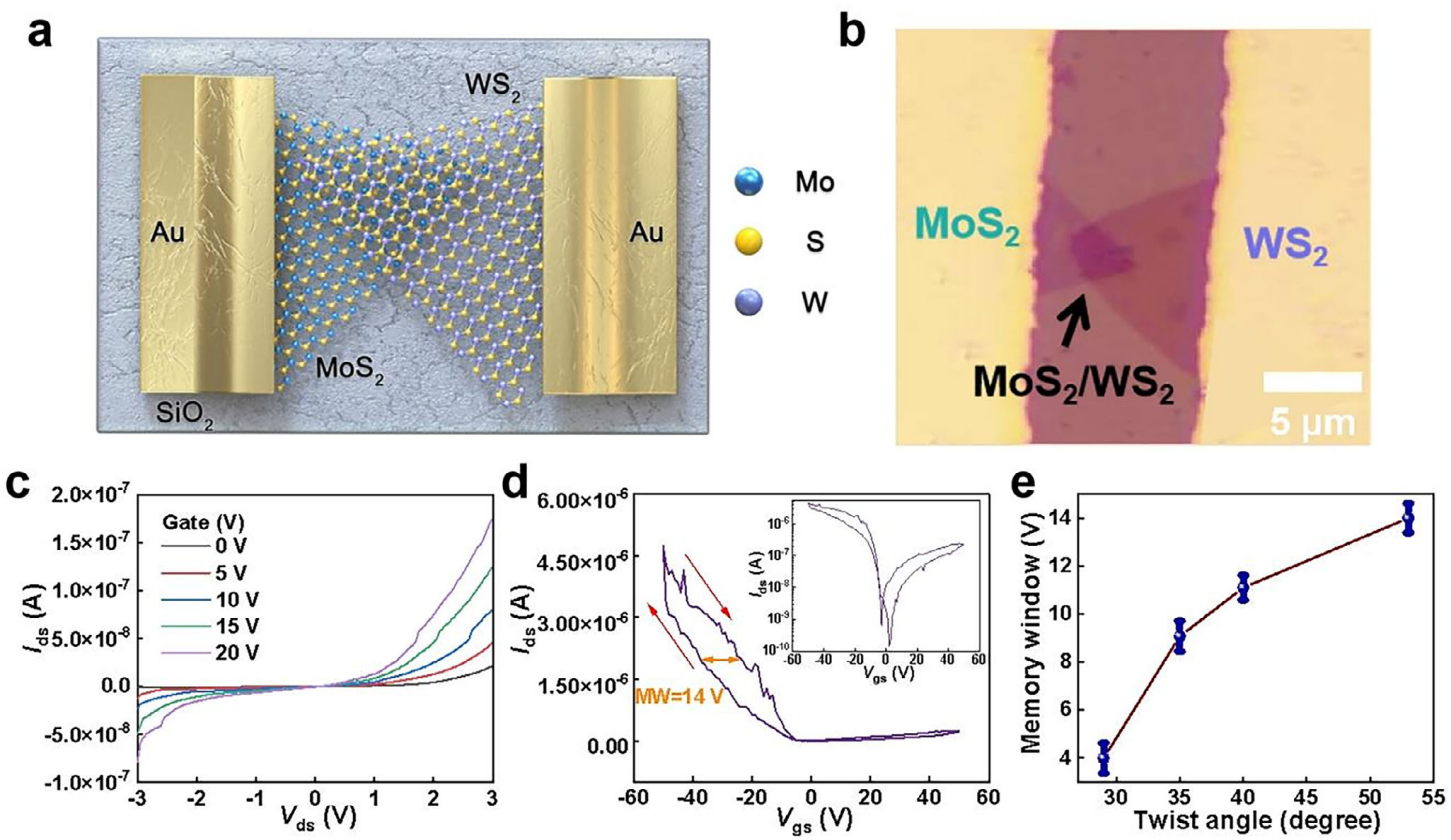
Electrical characterization of twisted MoS_2_/WS_2_ heterostructure based FeS‐FETs. a) Schematic diagram of FeS‐FETs composed of MoS_2_/WS_2_ heterostructure. b) Optical microscope image at a twisting angle of 53°. c) *I*_ds_‐*V*_ds_ curves of FeS‐FETs. d) *I*_ds_‐*V*_gs_ curves of FeS‐FETs and corresponding MW, and the inset shows the logarithmic result of *I*_ds_‐*V*_gs_ curve. e) MW of FeS‐FETs at different twisting angles.

2D ferroelectric materials have an extraordinarily important device application based on FeS‐FETs, where ferroelectric semiconductors are used as channel materials.^[^ ^23^ ^]^ As illustrated in **Figure** **5**a, the configuration of the FeS‐FETs with MoS_2_/WS_2_ heterostructure is schematically depicted. The gold electrode in contact with MoS_2_ is connected to the source, the gold electrode in contact with WS_2_ is connected to the drain. Figure 5b shows an optical microscope image at a twisting angle of 53°. Figure 5c depicts *I*_ds_‐*V*_ds_ curves of the FeS‐FETs with MoS_2_/WS_2_ heterostructure. The gate voltage is set to a range of 0∼20 V, with measurements taken at 5 V intervals. The observed nonlinear *I*_ds_‐*V*_ds_ characteristics suggest that gold forms a Schottky contact with the MoS_2_/WS_2_ heterostructure,^[^ ^24^ ^]^ and more FeS‐FETs devices can be displayed in Figure S10 (Supporting Information).

Figure 5d illustrates the *I*_ds_‐*V*_gs_ curve with the scanning voltage (‐50 to 50 V) applied to the gate and bias voltage of ‐1 V applied to the drain, and the inset shows the logarithmic result of *I*_ds_‐*V*_gs_ curve. The *I*_ds_‐*V*_gs_ curve exhibits that the *I*_ds_ value can be obviously changed under *V*_gs_, demonstrating gate‐tunable carrier concentration and transport within the channel. The symmetry breaking may give rise to the formation of an electric field at the interface, which in turn affects the charge distribution and transport. Unilateral energy band bending occurs at the interface, which induces a downward shift of the Fermi level toward the valence band edge. This electronic configuration facilitates surface hole accumulation through majority carrier enrichment, ultimately manifesting p‐type transistor behavior.^[^ ^25^ ^]^ The hysteretic of polarization as an intrinsic response mechanism of ferroelectric materials, which constitutes the origin of the hysteresis curve.^[^ ^26^ ^]^ The red arrow indicates the direction of hysteresis in the curve, and the clockwise hysteresis loop demonstrates that the MW is primarily determined by ferroelectric polarization rather than interface charge trapping.^[^ ^23^ ^]^ It can be clearly seen that the hysteresis in *I*_ds_‐*V*_gs_ curve, exhibiting a large MW of ∼14 V, which is larger than that of typical 2D ferroelectric materials (Figure S11, Supporting Information).^[^ ^27^, ^28^ ^]^ The MW has remained unchanged over 10^4^ cycles and 10^5^ s, as presented in Figure S12 (Supporting Information), exhibiting the excellent endurance/reliability of the devices. In addition, the FeS‐FETs have been constructed at varying twisting angles, and their ferroelectric MW values are quantified, as illustrated in Figure 5e. It can be observed that the MW increases gradually as the twisting angle rises. As shown in Figure 3e, the coercive voltage increases as the twisting angles are added. A large coercive voltage requires a large drive voltage to achieve the polarization reversal, thus expanding the MW.^[^ ^29^ ^]^ Moreover, the FeS‐FETs also offer a high mobility of ∼197 cm^2^ V^−1^ s^−1^.

## 3 Conclusion

In summary, the correlation between the ferroelectric properties and interlayer twist angle in bilayer MoS_2_/WS_2_ heterostructures is established. SHG and PFM characterizations reveal that by increasing the twist angles, the degree of symmetry breaking gradually decreases while the coercive voltage gradually increases. Theoretical calculations attribute this modulation to the evolution of distorted polar symmetry regions governed by Moiré patterns. Furthermore, FeS‐FETs comprising twisted ferroelectric MoS_2_/WS_2_ heterostructure have been constructed, which achieve the control of the electrical performance via the interlayer twist angle with large MW. This study provides that ferroelectric properties can be modulated by twisting angles, which will bring more new conceptualizations of sliding ferroelectric materials and emerging twistronics device applications.

## 4 Experimental Section

### Materials Preparation

The growth process was conducted in a two‐zone CVD system utilizing a 70‐inch quartz tube. The chemicals employed were molybdenum trioxide (MoO_3_, 99.999%), sodium chloride (NaCl, 99.9%), and sulfur (S, 99.9%) in powder form. The precursor powders were pre‐positioned within the furnace, thereby initiating the growth process. The temperature zone of MoO_3_ was heated to 700 °C at a rate of 10 °C/minute and maintained at this temperature for 15 minutes. During the growth process, Ar gas was employed as the carrier gas at a flow rate of 400 standard cubic centimeters per minute (sccm), the chamber pressure was maintained at atmospheric condition. Similarly, WO_3_ (99.999%), NaCl (99.9%), and S (99.9%) powder were precursors. Generally, the S powders and WO_3_ powders were placed on the upper stream and downstream of the furnace, respectively. Ar gas was purged into the chamber at 200 sccm, and the chamber pressure was maintained at atmospheric levels. The temperature was elevated to 780 °C and kept for 1 minute for growth. The as‐grown WS_2_ was transferred onto the MoS_2_ by a polydimethylsiloxane (PDMS)‐assisted transfer method. The polymethyl methacrylate (PMMA) solution was initially applied uniformly to the WS_2_ surface via spin coating. Once the film had undergone the drying process, it was then subjected to an etching procedure utilizing a sodium hydroxide solution. Subsequently, the etched film was transferred to PDMS. The lamination of thin films onto MoS_2_ was achieved through the 2D material transfer stage. Finally, the PMMA residue was removed using anisole solution.

### Characterizations of MoS_2_/WS_2_ Heterojunction

The Raman spectra were obtained using a confocal microscope system (WITec, Alpha 300) with a 532 nm laser at room temperature. The SHG response was also measured using an automatic pre‐dispersion compensation broadband‐tuned femtosecond oscillator (Coherent, Chameleon). The wavelength of the incident laser was 1064 nm. The sample thickness was characterized by AFM (Bruker, Multimode 8). The amplitude and phase loops of samples were measured by PFM (Bruker, Dimension Icon), the PFM tip was maintained at a constant direct current (DC) voltage while in contact with the MoS_2_/WS_2_ surface. The external electric field generated by DC voltage induced the ferroelectric polarization switching.

### Electrical Characterization of FETs

Gold thin film electrodes were employed in the construction of MoS_2_/WS_2_ based FETs. The electrodes were in direct contact with individual MoS_2_ and WS_2_, the central overlap was maintained. The *I*_ds_‐*V*_ds_ and *I*_ds_‐*V*_gs_ curves of FETs were measured using semiconductor analyzer with a probe state (Keithley 4200‐SCS).

### Theoretical Calculation

All first principles calculation work was carried out in the frame of density functional theory implemented in the software package VASP.^[^ ^30^ ^]^ The interaction between ions and electrons was described by the projector‐augmented wave method.^[^ ^31^ ^]^ The Perdew‐Berke‐Ernzerhof type of generalized gradient approximation was used to construct the electronic exchange‐correlation functionals,^[^ ^32^ ^]^ and the energy cutoff for the plane‐wave expansion of the wavefunction was set to 500 eV. In the calculation of the bilayer heterostructure material, the D3 method was considered to estimate the van der Waals effects. The Berry phase method was performed to calculate the polarization. To simulate the monolayer sample, the vacuum layer thickness was artificially set to 18 Å to isolate the interaction between the monolayer under periodic boundary conditions.

## Conflict of Interest

The authors declare no conflict of interest.

## Supporting information



Supporting Information

## Data Availability

The data that support the findings of this study are available from the corresponding author upon reasonable request.
