# Supplementary material for: Ferroelectric Properties of Bilayer MoS2/WS2 Heterostructure Modulated by Twist Angle
Source: Adv Sci (Weinh). 2025 Oct 8;12(48):e13738. doi: 10.1002/advs.202513738 (PMC12752589; doi:10.1002/advs.202513738)
Supplement: Supplementary file 1 — Supporting Information [file ADVS-12-e13738-s001.pdf]

Supporting Information

Ferroelectric properties of bilayer MoS_2/WS_2 heterostructure modulated by twist angle

Liyao Wang, Zhuopeng Xia, Xiaoyao Sun, Shiyao Xu, Guodong Sun, Yingying Zheng, Zhen Zhan, Enlong Li, Songhua Cai, Yuan Zhang, Jinzhu Zhao*, Wenwu Li*, Shuoguo Yuan*

L. Wang, X. Sun, G. Sun, Y. Zheng, S. Yuan
Faculty of Materials Science and Chemistry, China University of Geosciences, Wuhan 430074, China
E-mail: yuanshuoguo@cug.edu.cn
Z. Xia, J. Zhao
Guangdong Provincial Key Laboratory of Quantum Engineering and Quantum Materials, School of Physics, and Guangdong-Hong Kong Joint Laboratory of Quantum Matter, South China Normal University, Guangzhou 510006, China
E-mail: zhaojz@m.scnu.edu.cn
S. Xu, Y. Zhang
Department of Materials Science and Engineering, Southern University of Science and Technology, Shenzhen 518055, China
Z. Zhan, S. Cai
Department of Applied Physics, The Hong Kong Polytechnic University, Hong Kong 999077, China
E. Li, W. Li
Shanghai Frontiers Science Research Base of Intelligent Optoelectronics and Perception, Institute of Optoelectronics, Department of Material Science, Fudan University, Shanghai 200433, China
E-mail: liwenwu@fudan.edu.cn
J. Zhao
Center for Computational Science and Engineering, Southern University of Science and Technology, Shenzhen 518055, P. R. China; National Laboratory of Solid State Microstructures, Nanjing University, Nanjing 210093, P. R. China
S. Yuan
Shenzhen Institute, China University of Geosciences, Shenzhen 518057, China

Supplementary Text

1. Sample fabrication and structural characterization of MoS_2/WS_2 heterostructure

The growth of monolayer MoS_2 and WS_2 was conducted by chemical vapor deposition (CVD) method in a dual-temperature zone tube furnace, as shown in **Figure S1**a. During the CVD growth stage, the length of the tube furnace was 70-inch, with an outer diameter of 100 mm and a thickness of 5 mm. To facilitate the growth process of both materials, the following substances were employed as precursors, reactants, and fluxes, respectively: S powder, MoO_3/WO_3, and NaCl. The Ar gas was employed as the carrier gas. Two distinct quartz boats were positioned within the two temperature zones, with the quartz boat containing the S powder situated in the upstream position. The silicon substrate was positioned with its surface facing downwards on top of the downstream quartz boat.

As illustrated in Figures S1b,c, following a series of parameter optimization, the optimal conditions for the growth of MoS_2 were as follows: an initial dosage of MoO_3 (10 mg), NaCl (5 mg), S (0.4 g), and a carrier gas flow rate of 400 standard cubic centimeters per minute (sccm). The upper-temperature zone was heated to 220 °C for 68 minutes, while the lower zone was heated to 700 °C for the same duration. The temperature was maintained at a warm state for 15 minutes before being allowed to naturally cool down to room temperature. To facilitate the growth of WS_2, the most refined material was cultivated using an initial dosage of WO_3 (9 mg) + NaCl (3 mg), S (0.4 g), and a carrier gas flow rate of 200 sccm. This was achieved by gradually increasing the upper temperature zone to 200 °C during the 75 minutes period and simultaneously ramping up the lower zone to 780 °C for an identical duration. The 1 minute holding period was initiated, after which the material was allowed to naturally cool to room temperature. Before each growth cycle, the gas was subjected to three cycles of Ar gas washing and the SiO_2/Si substrate underwent sonication to ensure the sample's purity and the quality of the growth.

Optical microscope images of CVD-grown samples were presented in Supplementary

Figures S1d,e. Subsequently, the WS_2 was etched onto the MoS_2 wafer by the wet transfer method, the overall flow chart is shown in Figure 1a in the main text. Using a spin coater, the prepared polymethyl methacrylate (PMMA) solution (PMMA particles dissolved in anisole, with a concentration of 6%) was dripped onto monolayer WS_2. Low-speed spin-coating (4000 rpm, 10 s) was applied to ensure uniform thickness, followed by high-speed spin-coating (5000 rpm, 10 s) to remove impurities. After this, the sample was baked at 90 °C for 10 minutes to ensure adhesion of the material to the film. It was then etched in a NaOH solution (2 g NaOH + 50 ml deionized water) until the film spontaneously detached completely. The detached film was retrieved, and its back side (non-material side) was attached to polydimethylsiloxane (PDMS). Using a two-dimensional (2D) material transfer stage, the front side (material side) was transferred onto a monolayer MoS_2 substrate. During the transfer process, the twist angle of the heterostructure was precisely controlled by adjusting the orientation of the substrate. Subsequently, heating was applied to reduce the adhesion of PDMS, allowing the film to conform naturally to the substrate. After transfer, the sample was left undisturbed for 12 hours to ensure complete adhesion. Finally, it was immersed in anisole and acetone for 1 hour each to remove residual PMMA and PDMS, resulting in a clean and twisted MoS_2/WS_2 heterostructure.

As shown in **Figure S2**, the scanning transmission electron microscopy (STEM) image reveals the bilayer structure of the MoS_2/WS_2 with well-defined interface sharpness, and no discernible wrinkles or damage are observed at the interface. Meanwhile, energy-dispersive x-ray spectroscopy (EDS) result confirms distinct layering of W elements in the upper layer and Mo elements in the bottom layer, along with a uniform distribution of S elements across both layers, effectively ruling out disordered stacking and ensuring interfacial quality.[S1]

2. SHG response of MoS_2/WS_2 heterostructure

The second-harmonic generation (SHG) spectroscopy was used to characterize the crystal symmetry dependence and the symmetry of twisted structures. From **Figure S3**, we can

observe that there are significant SHG signals in both parallel and perpendicular polarization configurations, with an angle discrepancy of 30°. As shown in Figure 2 in the main text, the twisted heterostructure exhibits rhombohedral symmetry characteristics and non-centrosymmetric structural features. The SHG is employed to ascertain structural information about MoS_2/WS_2 heterostructure, given that the method is highly sensitive to crystal symmetry, and the stacking pattern of the material. **Figure S4** shows the SHG signals generated at different twisting angles. This observation aligns with the theoretical calculation previously discussed. Moreover, it can be demonstrated that the degree of symmetry breaking decreases as the twisting angle gradually increases.

3. PFM characterization of MoS_2/WS_2 heterostructure

The piezoresponse force microscopy (PFM) is employed for the assessment of ferroelectric characteristics in MoS_2/WS_2 heterostructure. The PFM measurements were conducted on different samples of MoS_2/WS_2 heterostructure with varying twisting angles. As illustrated in **Figure S5**, the hysteresis phase loops exhibit a 180° phase switching and the ferroelectric butterfly behavior, displaying out-of-plane ferroelectricity. Subsequently, the coercive voltage values of the ferroelectric polarization have been quantified, revealing a discernible pattern with the change of twisting angle.

To rule out the charge effects, we have removed the bias voltage and measured the PFM phase and butterfly curves of MoS_2/WS_2 heterostructure over three days (72 hours) to examine the stability of the ferroelectric polarization state. As shown in **Figures S6**a,b, the two curves remain stable after the removal of the extrinsic bias, with no significant change in their signals observed over 72 hours period. This non-volatile retention of the polarized state excludes the possibility of electrostatic charging, confirming the characteristic of ferroelectricity.[S2] Concerning the test of ferroelectric endurance, we will demonstrate the result in the ferroelectric semiconductor field-effect transistors (FeS-FETs), the FeS-FETs maintain relatively stable of the ferroelectric memory window (MW) over multiple cycles.[S3]

These results convincingly confirm that the observed phenomena originate from intrinsic ferroelectric switching rather than extrinsic artifacts.[S4]

4. Atomic structures used in theoretical calculations

Figure S7 shows the atomic structure of untwisted stacking configuration, and **Figure S8** exhibits different twist angles of the atomic structure. In the main text, we have presented the potential-energy changes during sliding at a twisting angle of 21°, and also investigated other angles. Energy minima occur when the material is not sliding, and at 1/3 and 2/3 unit-cell displacements. Furthermore, as illustrated in the atomic structure diagram, alterations in the twisting angles result in significant changes to the overall symmetry.

To investigate the role of kinetic barriers for polarization switching and interlayer charge transfer, as shown in **Figure S9**a, except for the un-twisted case, the total energy for the rest configuration shows relatively similar values. Based on these, we have estimated the energetical barrier is below 10 meV/f.u. in most cases.[S5, S6] Besides, we have systematically performed Bader charge analysis at discrete twist angles (Figure S9b). These data reveal that, as θ increases, the net positive and negative charges accumulated on the top and bottom layers decrease concurrently, suppressing the out-of-plane polarization and driving the total energy upward. At θ = 0°, the top and bottom layers harbor equal-magnitude, opposite-sign net charges, engendering a pronounced out-of-plane polarization; with increasing θ, this charge asymmetry diminishes, concomitantly attenuating the polar component and affording qualitative accord with the polarization suppression upon twisting.

5. MoS_2/WS_2 heterostructure based FeS-FETs

The FeS-FETs have been constructed utilizing the MoS_2/WS_2 heterostructure. The I_{ds}-V_{ds} curves are shown in **Figures S10**a-c. The curves change with different gate biases, demonstrating the influence of the gate voltage on the formation of the transistor conducting channel and the movement of electrons. Figures S10d-f illustrates the I_{ds}-V_{gs} transfer characteristic curve, and the mobility is calculated in accordance with the following formula:

$$\mu = \frac{L}{W} \times \frac{1}{C_i} \times \frac{1}{V_{ds}} \times \frac{dI}{dV_{gs}}$$

Where L and W denote the length and width of the channel, C_i denotes the capacitance, V_{ds} denotes the drain voltage, respectively. The mobility of different devices with around 107 $cm^2/V{\cdot}s$, 124 $cm^2/V{\cdot}s$ and 132 $cm^2/V{\cdot}s$ can be obtained.

Compared to the MW values of other 2D ferroelectric transistors,[S7-S14] the twisted MoS_2/WS_2 ferroelectric transistors with large MW value of ~14 V, as shown in **Figure S11**. The MW of the FeS-FETs remains almost unchanged over the retention time from 10^1 to 10^5 s, corroborating the excellent endurance of the device (**Figure S12**a). In addition, the MW remains stable with increasing number of cycles, indicating good reliability characteristics of the device (Figure S12b). Simultaneously, the excellent cycling and clockwise hysteresis indicate that the MW primarily originates from the polarization switching of the ferroelectric layer.[S15-S17]

References

[S1] L. Rogée, L. Wang, Y. Zhang, S. Cai, P. Wang, M. Chhowalla, W. Ji, S. Lau, *Science* 2022, **376**, 973.

[S2] W. Mu, C. Ke, C. Huangfu, J. Dong, Y. Zhou, J. Zheng, S. Yue, J. Li, S. Liu, L. Jiao, *Adv. Mater.* 2025, **37**, 2504941.

[S3] Q. Li, S. Wang, Z. Li, X. Hu, Y. Liu, J. Yu, Y. Yang, T. Wang, J. Meng, Q. Sun, D. W. Zhang, L. Chen, *Nat. Commun.* 2024, **15**, 2686.

[S4] R. Bian, R. He, E. Pan, Z. Li, G. Cao, P. Meng, J. Chen, Q. Liu, Z. Zhong, W. Li, F. Liu, *Science* 2024, **385**, 57.

[S5] X. Luo, X. He, R. Wang, H. Xiang and J. Zhao, *Nano. Lett.* 2025, **25**, 10145.

[S6] X. Chen, J. Zhao, P. Ghosez, *Phys. Rev. B* 2024, **110**, 245302.

[S7] P. Singh, S. Baek, H. H. Yoo, J. Niu, J. H. Park, S. Lee, *ACS Nano* 2022, **16**, 5418.

[S8] T. Yang, B. Liang, H. Hu, F. Chen, S. Ho, W. Chang, L. Yang, H. Lo, T. Kuo, J. Chen, P. Lin, K. Simbulan, Z. Luo, A. Chang, Y. Kuo, Y. Ku, Y. Chen, Y. Huang, Y. Chang, Y. Chiang, T. Lu, M. Lee, K. Li, M. Wu, Y. Chen, C. Lin, Y. Lan, *Nat. Electron.* 2024, **7**, 29.

[S9] X. Wang, C. Zhu, Y. Deng, R. Duan, J. Chen, Q. Zeng, J. Zhou, Q. Fu, L. You, S. Liu, J. Edgar, P. Yu, Z. Liu, *Nat. Commun.* 2021, **12**, 1109.

[S10] C. Song, D. Kim, S. Lee, H. Kwon, *Adv. Sci.* 2024, **11**, 2308588.

[S11] J. Liao, W. Wen, J. Wu, Y. Zhou, S. Hussain, H. Hu, J. Li, A. Liaqat, H. Zhu, L. Jiao, Q. Zheng, L.

Xie, *ACS Nano* 2023, **17**, 6095.

[S12] S. Wang, L. Liu, L. Gan, H. Chen, X. Hou, Y. Ding, S. Ma, D. Zhang, P. Zhou, *Nat. Commun.* 2021, **12**, 53.

[S13] W. Han, X. Zheng, K. Yang, C. Tsang, F. Zheng, L. Wong, K. Lai, T. Yang, Q. Wei, M. Li, W. Io, F. Guo, Y. Cai, N. Wang, J. Hao, S. Lau, C. Lee, T. Ly, M. Yang, J. Zhao, *Nat. Nanotechnol.* 2023, **18**, 55.

[S14] H. Xiang, Y. Chien, L. Li, H. Zheng, S. Li, N. Duong, Y. Shi, K. Ang, *Adv. Funct. Mater.* 2023, **33**, 2304657.

[S15] L. Zhao, Y. Liang, J. Ma, Z. Pan, X. Liu, M. Yang, Y. Sun, W. Gao, B. Li, J. Li, N. Huo, *Adv. Funct. Mater.* 2023, **33**, 2306708.

[S16] K. Kim, S. Song, B. Kim, P. Musavigharavi, N. Trainor, K. Katti, C. Chen, S. Kumari, J. Zheng, J. Redwing, E. Stach, R. Olsson, D. Jariwala, *ACS Nano* 2024, **18**, 4180.

[S17] M. Si, A. Saha, S. Gao, G. Qiu, J. Qin, Y. Duan, J. Jian, C. Niu, H. Wang, W. Wu, S. Gupta, P. Ye, *Nat. Electron.* 2019, **2**, 580.

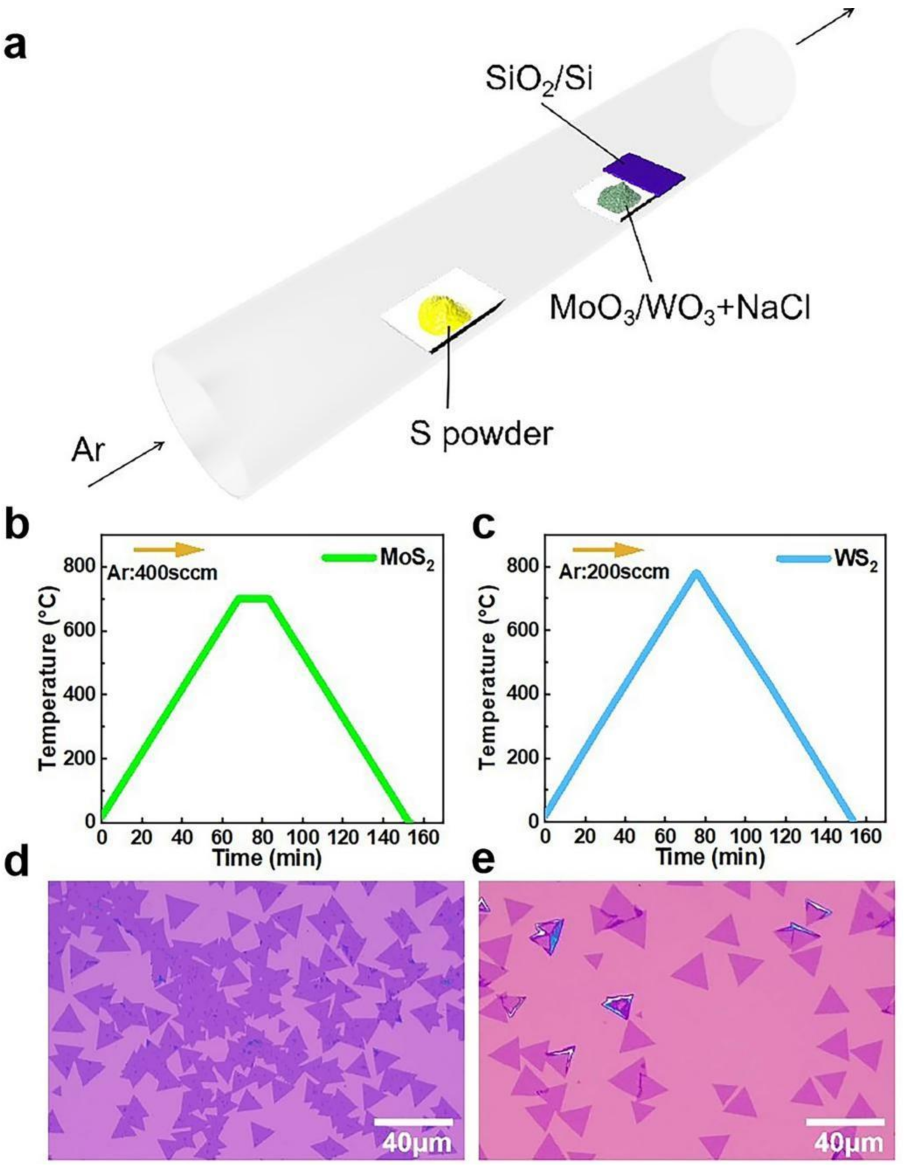

Figure S1. CVD growth of MoS_2 and WS_2. a) Schematic diagram of CVD growth of MoS_2/WS_2. b,c) Experimental growth parameter corresponding to the growth of monolayer MoS_2 (b) and WS_2 (c). d,e) Optical microscope images of CVD-grown MoS_2 (d) and WS_2 (e).

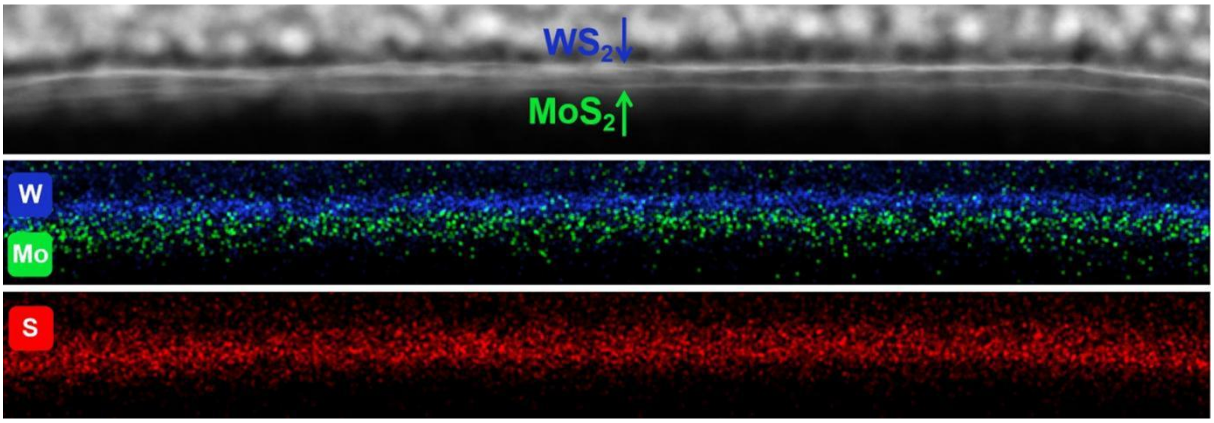

Figure S2. The interface of the twisted MoS_2/WS_2 heterostructure was characterized by STEM and EDS elemental mapping.

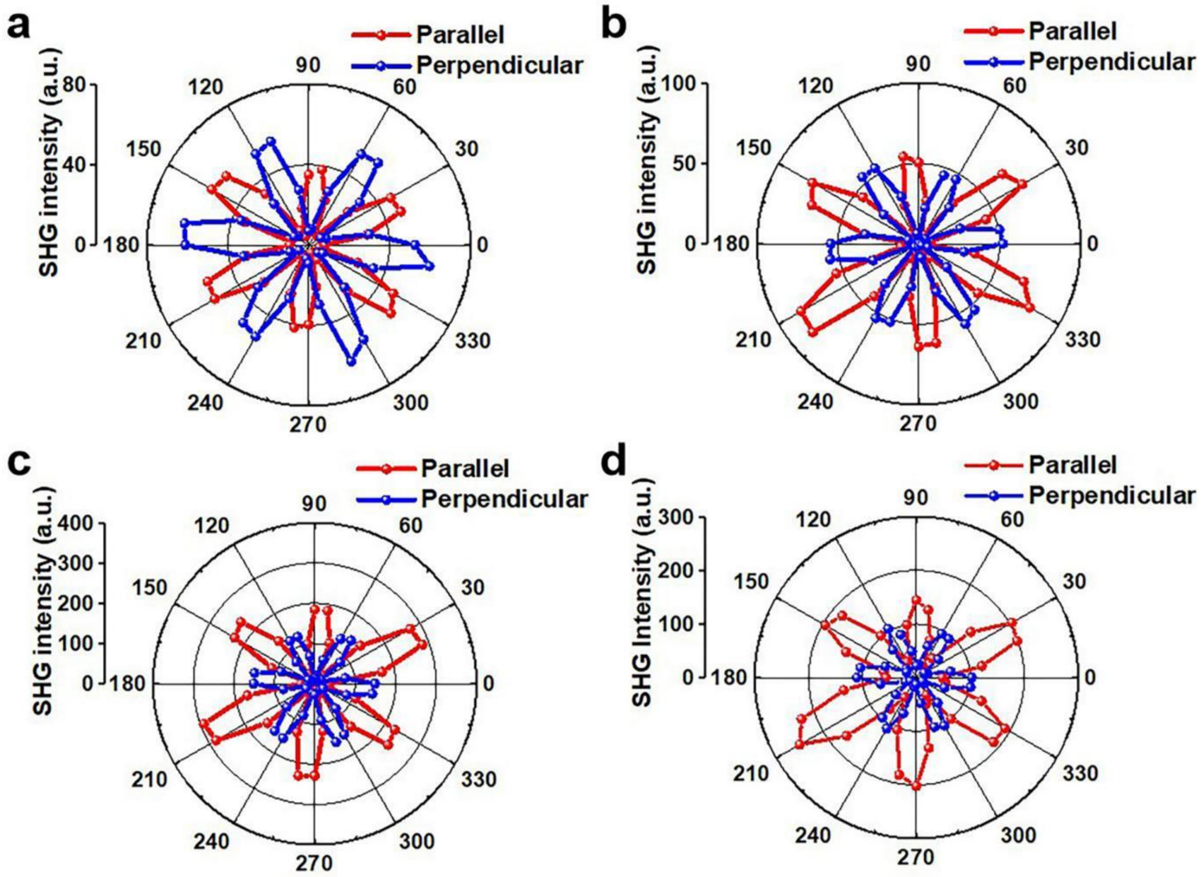

Figure S3. SHG polarization images as a function of different twist angles. a) 7°, b) 14°, c) 25°, and d) 29°.

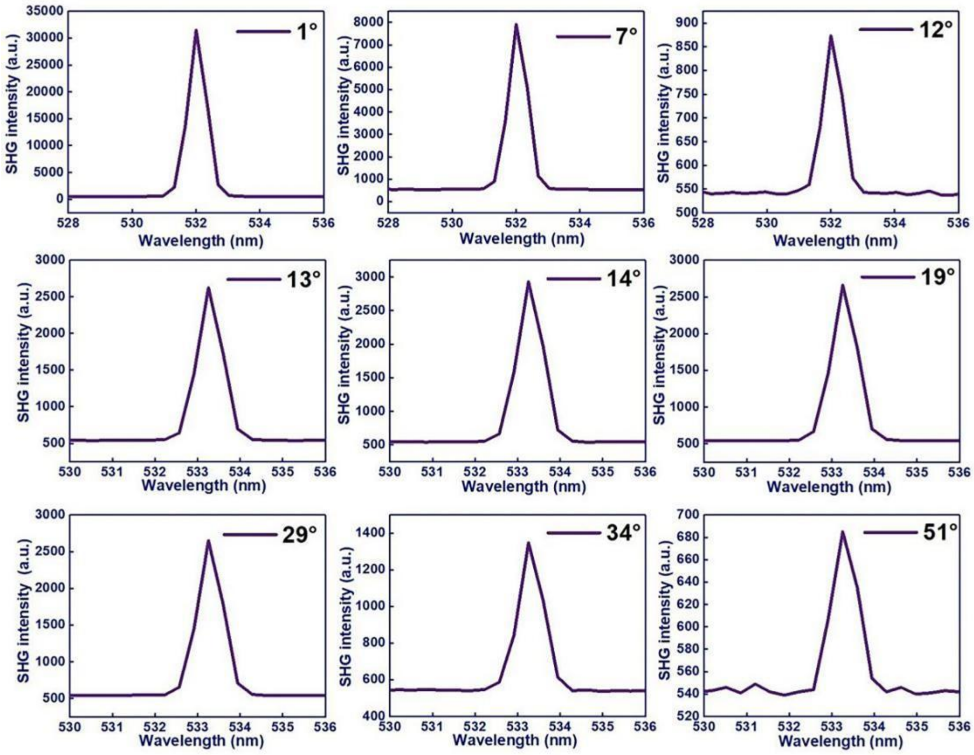

Figure S4. SHG intensity curves with different twist angles. The pulsed laser was made with a wavelength of 1064 nm, with its response occurring at 532 nm.

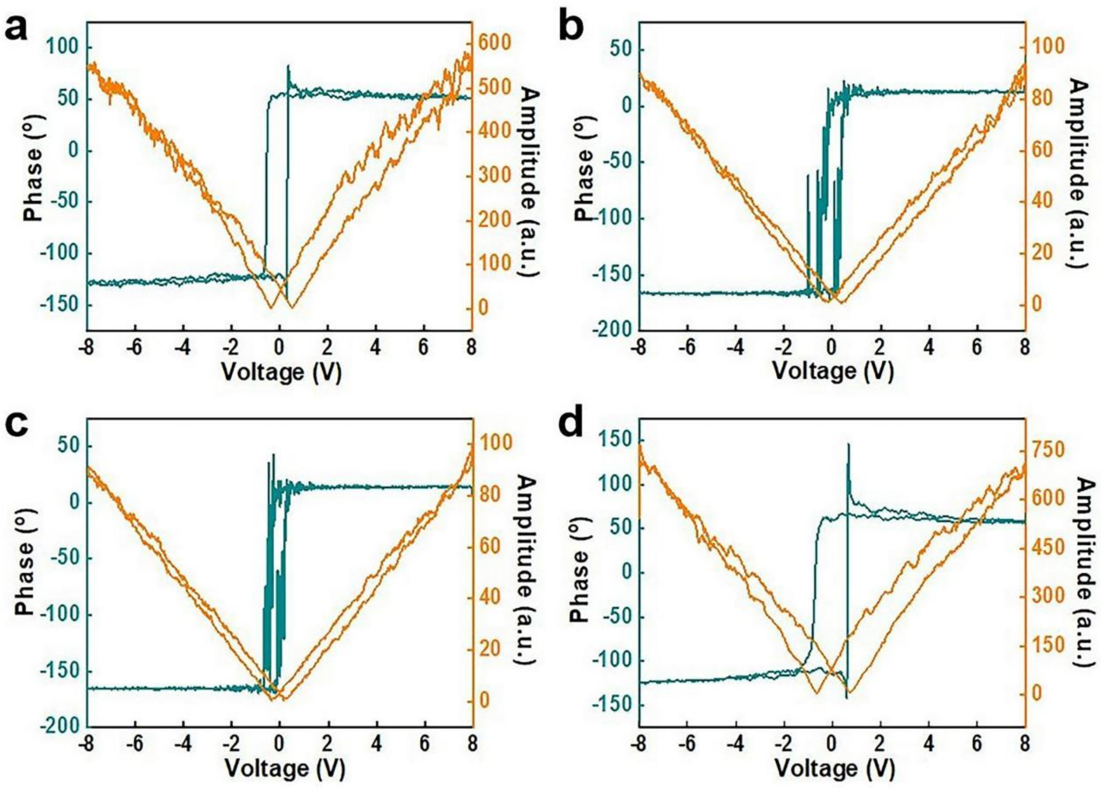

Figure S5. The phase and amplitude hysteresis loops of MoS_2/WS_2 heterostructure with different twist angles. a) 7°, b) 13°, c) 19° and d) 34°.

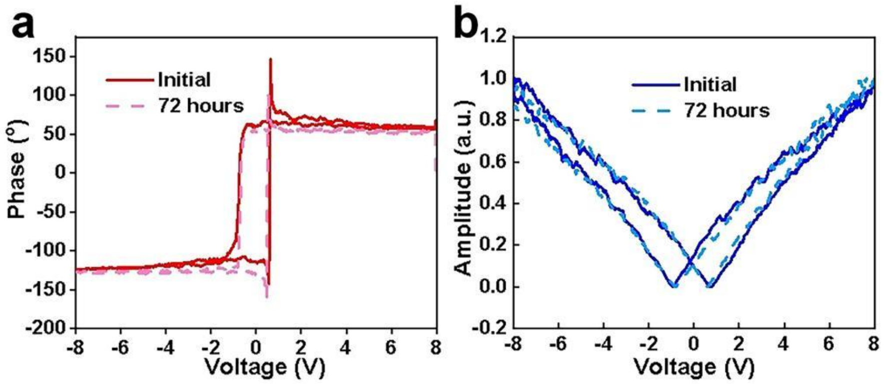

Figure S6. Comparison of the a) phase and b) amplitude hysteresis loops of MoS_2/WS_2 heterostructure with initial state and after 72 hours.

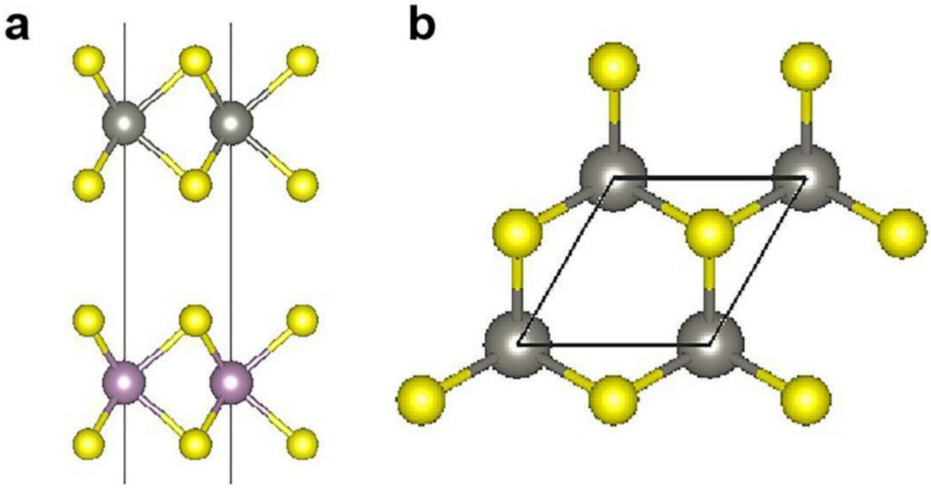

Figure S7. Parallel stacked atomic structure. a) The side view of the MoS_2/WS_2 heterostructure. The upper layer is composed of WS_2, while the lower layer is composed of MoS_2. b) The top view of the MoS_2/WS_2 heterostructure. The purple, black, and yellow colors denote the Mo atom, W atom, and S atom.

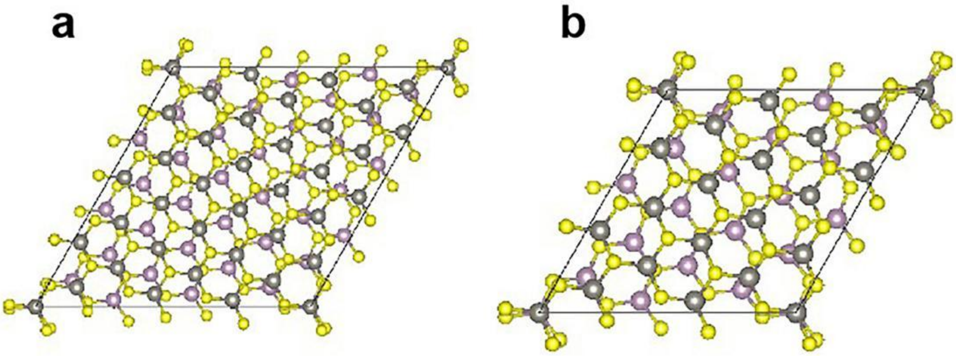

Figure S8. The top view of a Moiré superlattice with twist angles. a) 9°, b) 13° with all angles pointing in the same direction. The purple, black, and yellow colors denote the Mo atom, W atom, and S atom.

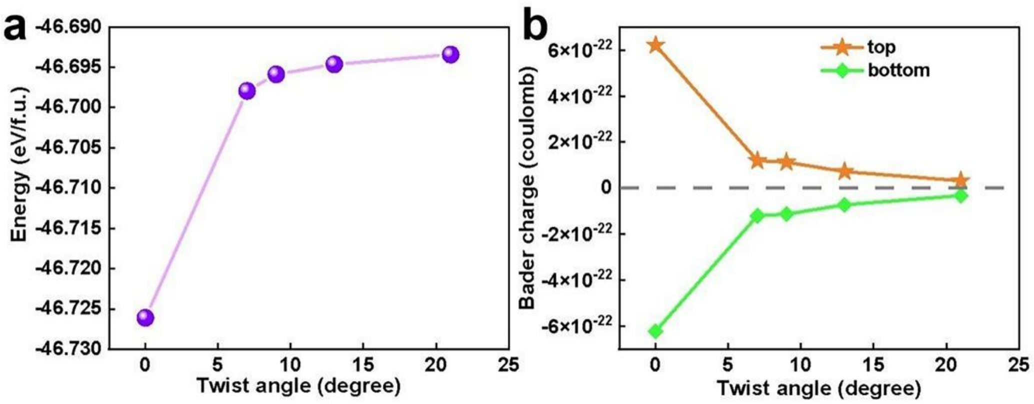

Figure S9. a) Single-point energies of the untwisted and four discrete twist-angle configurations. b) Corresponding Bader charges on the top and bottom layers.

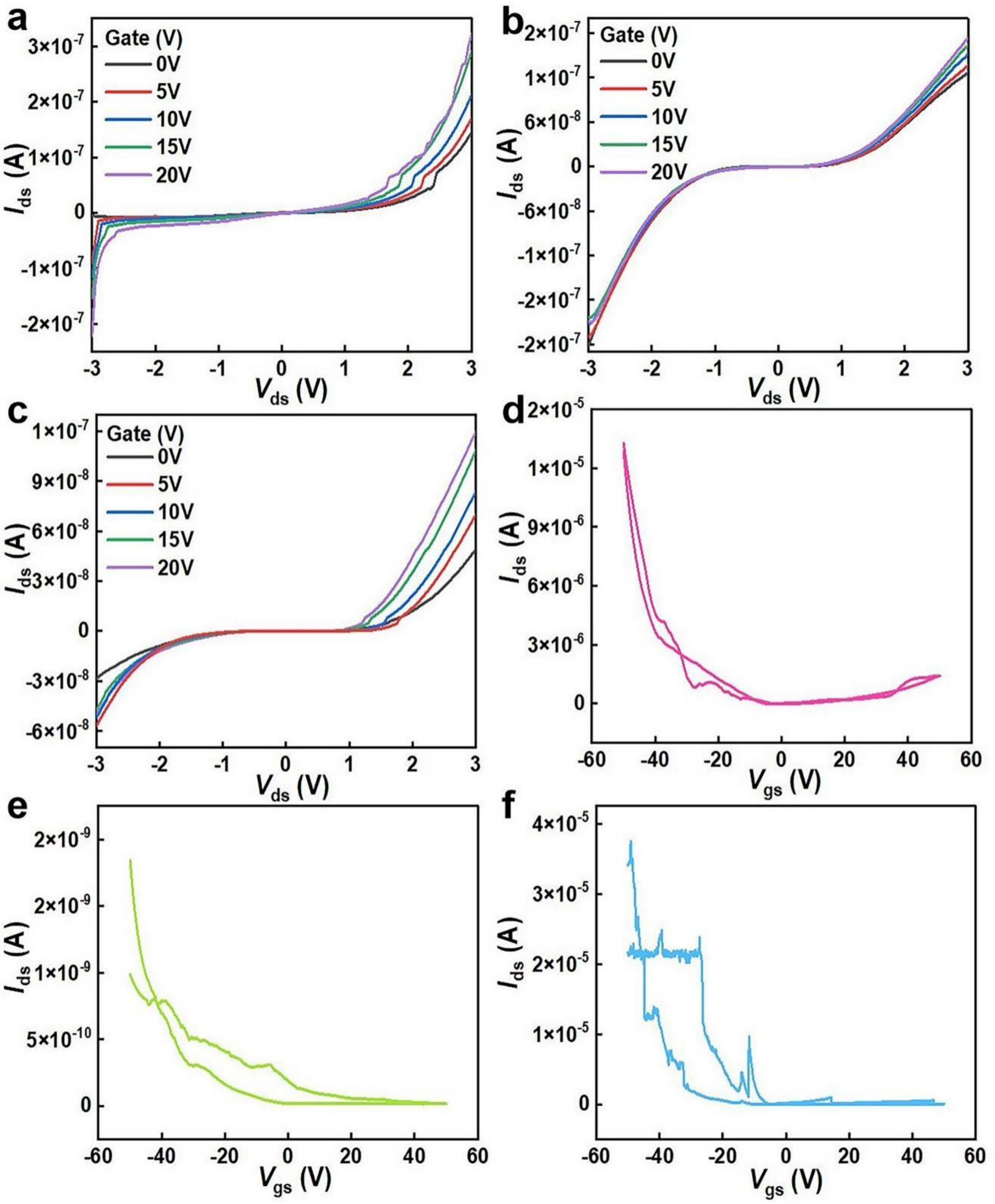

Figure S10. Electrical characterizations of twisted MoS_2/WS_2 based FeS-FETs at different twist angles. a-c) I_{ds}-V_{ds} curves of FeS-FETs: a) 29°, b) 35°, c) 40°. d-f) I_{ds}-V_{gs} curves of FeS-FETs: d) 29°, e) 35°, f) 40°.

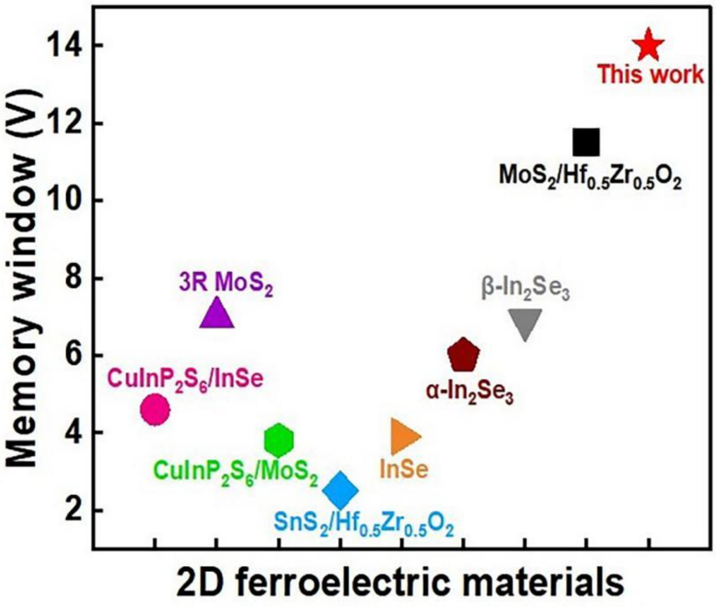

Figure S11. Comparison of the reported MW values for the typical 2D materials based ferroelectric transistors: $CuInP_2S_6$/InSe[S7], 3R-MoS_2[S8], $CuInP_2S_6$/MoS_2[S9], SnS_2/$Hf_{0.5}Zr_{0.5}O_2$[S10], InSe[S11], α-In_2Se_3[S12], β-In_2Se_3[S13], and MoS_2/$Hf_{0.5}Zr_{0.5}O_2$[S14].

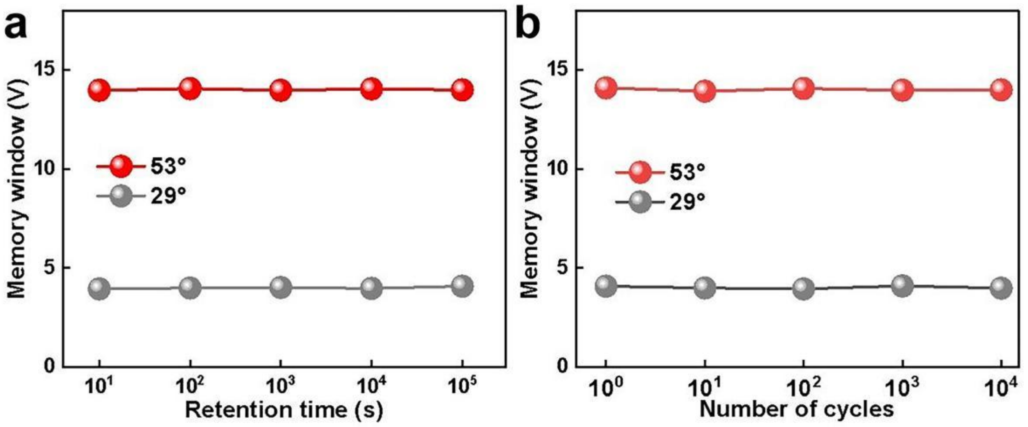

Figure S12. Endurance/reliability data at the twist angles of 29° and 53°. MW under different a) retention times and b) number of cycles.
